# Stream grazers determine their crawling direction on the basis of chemical and particulate microalgal cues

**DOI:** 10.7717/peerj.503

**Published:** 2014-08-21

**Authors:** Izumi Katano, Hideyuki Doi

**Affiliations:** 1 Aqua Restoration Research Center, Public Works Research Institute, Kakamigahara, Japan; 2 School of Human Science and Environment, University of Hyogo, Himeji, Japan; 3 Institute for Sustainable Sciences and Development, Hiroshima University, Higashi-Hiroshima, Japan

**Keywords:** Cue communication, Prey, Herbivore, Primary producer, Predator, Caddisfly

## Abstract

This study aimed to determine the association between herbivore behavior and cues from producers. We used stream grazer *Glossosoma* larvae and determined their crawling direction in relation to chemical and visual cues from microalgae. The experimental treatments included control (no cue), particulate (chemical and particulate cues), and dissolved (chemical cue) cues from microalgae. The experimental water samples were randomly placed into either arm of a Y-shaped channel, and the crawling direction of the grazers was determined. Although the grazers crawled toward the arm containing either particulate or dissolved cues, they preferred the arm with particulate cues. This suggested that grazers responded well to both particulate (i.e., drifting algal cells) and chemical (algal smell) cues, and that particulate cues were more important for foraging. In natural habitats, grazers detect cues from producers and change their behaviors to maintain a balance between top-down and bottom-up cues.

## Introduction

Chemical and visual cues play important roles in species interactions (Lima, 1998a; Lima, 1998b; Burks & Lodge, 2002; Van Donk, Ianora & Vos, 2011). Numerous studies have established that these cues are also important in predator–prey interactions (e.g., Turner & Montgomery, 2003; Takahara et al., 2012; Takahara et al., 2013). Predators can be attracted to food patches by recognizing cues from animal prey (Lonnstedt, McCormick & Chivers, 2012; McCoy et al., 2012).

The detection of cues also occurs between predators (herbivores) and primary producers. Many studies have shown that morphological changes occur in primary producers on the basis of cues from predators (Lürling & Von Elert, 2001; Selander et al., 2011; Van Donk, Ianora & Vos, 2011). However, few studies have investigated the herbivore responses to producer cues, and these studies have been limited to consumption (Poulet & Marsot, 1978; DeMott, 1986), habitat use (Doi, Katano & Kikuchi, 2006), and foraging behavior (Katano, Doi & Oishi, 2009).

In stream ecosystems, grazers need to be able to respond to patchily distributed periphyton (Biggs, 1996; Hollingsworth & Vis, 2010). Various grazers perform area-restricted searches (Krebs, 1978) to obtain abundant food resources (Hart, 1981; Kohler, 1984). Caddisfly and mayfly grazers utilize alternative behaviors depending on periphyton abundance; they move rapidly until they arrive at a patch having abundant periphyton and then move slowly within the patch (the former is an extensive form of search, and, the latter, intensive, Poff & Ward, 1992; Katano et al., 2005; Hoffman et al., 2006).

Doi, Katano & Kikuchi (2006) and Katano, Doi & Oishi (2009) hypothesized that cues from periphyton (i.e., microalgal cues) mediate the interaction between grazers and periphyton. The presence of microalgal cues was confirmed by changes in the habitat use and searching behavior of *Glossosoma* larvae that detected and responded to the microalgal cues. However, they did not determine whether the microalgal cues were chemical, visual, or tactile. Diatoms are generally known to drift from periphyton mats (Bothwell et al., 1989; Stevenson & Peterson, 1991), and such drifting diatoms might function as microalgal cues for herbivores. Diatoms are known to drift more at night than during the day (Bothwell et al., 1989), and Doi, Katano & Kikuchi (2006) reported that the habitat use of *Glossosoma* larvae in patches with abundant periphyton was greater at night than during the day. On the basis of this finding, we speculated that the larvae might detect microalgae (i.e., flowing microalgal cells) via particulate cues, (hence, hereafter, we use particulate cues for microalgal cues). However, this hypothesis has never been tested, although knowledge about cue mechanisms (chemical or particulate) is important for completely understanding the interaction of cues between producers and stream herbivores.

In this study, we performed a laboratory experiment to determine whether microalgal cues (chemical or particulate or both) play an important role in the interaction between periphyton and grazers by using the larvae of caddisfly grazer, *Glossosoma* sp. (Trichoptera: Glossosomatidae). The genus *Glossosoma* includes grazer species that feed on periphyton. The larvae of these species build dome-shaped sand cases and crawl on the surfaces of stones in riffles (Feminella & Hawkins, 1995; Merritt & Cummins, 1996). *Glossosoma* larvae are often the dominant grazers in Japanese streams with hard substrates (Doi & Katano, 2008).

## Materials and Methods

### Collection of *Glossosoma* larvae and experimental waters

In June 2006, we collected 60 cobbles to acquire periphyton, *Glossosoma* larvae (2.3 ± 0.9 mg dry mass), and river water from riffles in the Agi-gawa River, a tributary of the Kiso-gawa River system, Gifu Prefecture, Japan (35°26′49″N, 137°25′12″E). Periphyton were brushed off the cobbles and placed in a container with 1 L of surface river water. The container and living larvae were transported in an aerated cooler to the laboratory. No specific permits were required for the field studies described, because the location was not privately owned or protected, and the field studies did not involve capturing endangered or protected species.

In the laboratory, we prepared three types of experimental water treatments: filtered river water as a control, dissolved microalgal cue water, and particulate microalgal cue water. For the control, 60 L of Agi-gawa river water was filtered through a GA-100 glass filter (Toyo-roshi Co., Tokyo, Japan; pore size, 1 µm). Particulate microalgal cue water was obtained by adding half of the well-mixed periphyton suspension water into 60 L of filtered river water. For the dissolved microalgal cue water, the other half of the well-mixed periphyton suspension was passed through a glass filter (GA-100; Toyo-roshi Co., Tokyo, Japan; pore size, 1 µm) and added to 60 L of filtered river water.

In the experiment, we assumed the materials in the experiment water as follows. The control water only contained the background levels of chemical cues and no particulates. The dissolved water contained the extracellular cues from periphyton, such as the chemicals from the algal cells and bacteria, but did not contain the algal cells due to the small pore size of the filter (1 µm). The particulate water contained all materials from periphyton including the cells and chemicals.

The abundance of microalgal cells in each water treatment was estimated by measuring chlorophyll *a* (Chl *a*) concentration. If the experimental water contained only chemical cues and not algal cells, the Chl *a* concentration would be negligibly low. Well-mixed experimental water (100 mL per treatment) was filtered through a glass filter (GA-100; Toyo-roshi Co., Tokyo, Japan; pore size, 1 µm). The filter was then cut into small pieces to extract chlorophyll and placed into a vial, each containing 20 mL of 99.5% ethanol. The vials were preserved in the dark at 4 °C for 24 h; subsequently, the extracted pigments were measured using a MPS-2000 spectrophotometer (Shimadzu Co., Japan). The Chl *a* contents were determined according to the method of UNESCO (1966), and then the abundance of microalgae in the experimental water was assessed (mg Chl *a* m^−3^).

### Experiment channel

**Figure 1:**
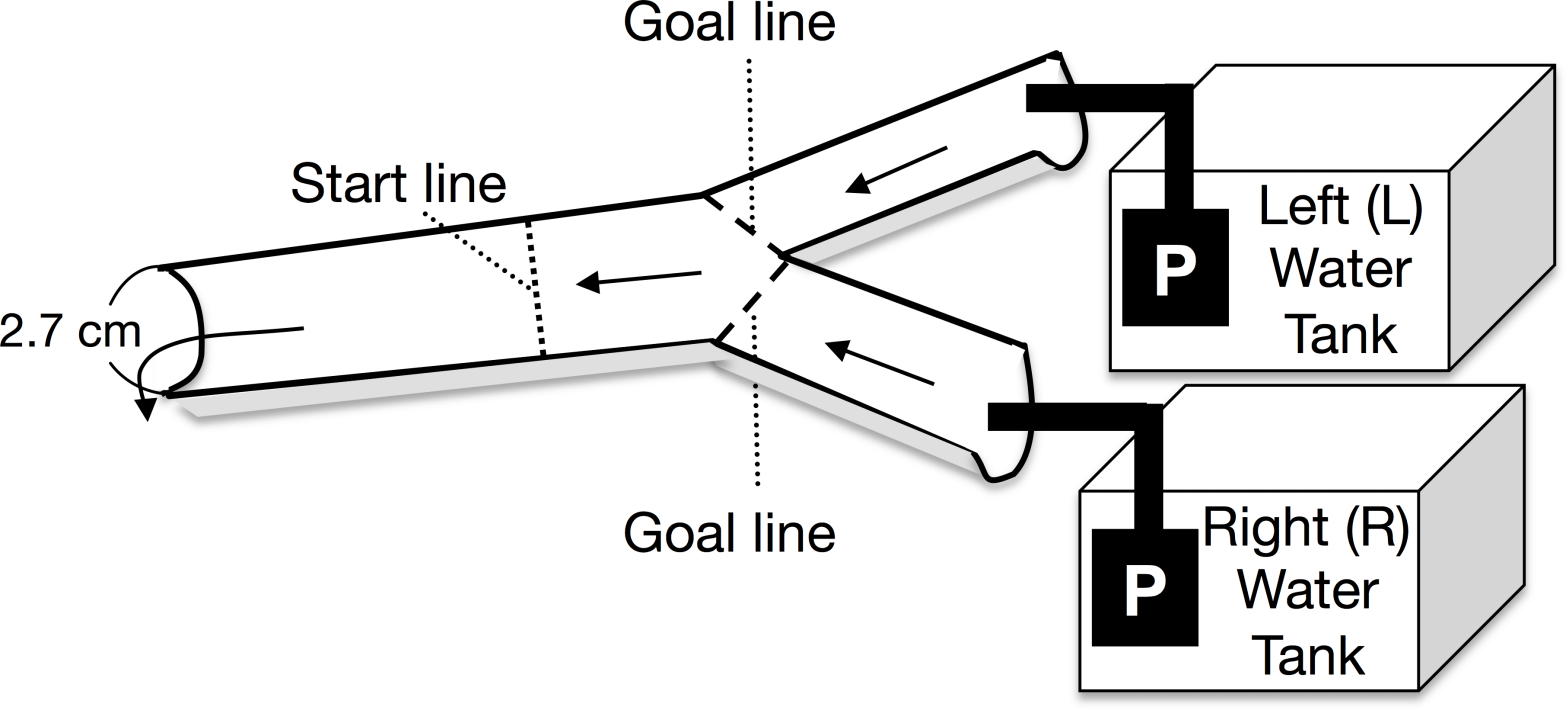
Top view of the experimental channel. Arrows and P show the water flows and water pumps, respectively.

A Y-shaped, one-way experimental channel (branched zone: 20 cm long × 2.5 cm wide × 0.5–0.7 cm water depth; experimental zone: 25 cm long × 2.7 cm wide × 0.7–1 cm water depth) was used for the experiment (Fig. 1). Two types of experimental water were randomly placed in either the left or right arm of the water tank. The current velocity at the start line was maintained at 8.3 ± 1.8 cm s^−1^ (mean ± 1 SD, *n* = 48) by using two aquarium water pumps on the water tanks (Rio-1100; Kamihata Fish Industry Co., Himeji, Japan). The experimental water flowed through the Y-shaped channel and was not recycled. The discharge from the left and right pumps to the branched channels ranged from 3.7 to 8.0 mL s^−1^ and from 3.9 to 8.4 mL s^−1^ respectively. Analysis of covariance (ANCOVA) showed that the discharge from the left and right pumps was not significantly different (*F* = 0.42, *p* = 0.40, *n* = 48); further, there was no significant interaction between the discharge and the water in the tanks (*F* = 0.42, *p* = 0.52). During the experiment, discharge from both the pumps to the branched (i.e., left and right) channels was repeatedly measured. An increase in the water temperature in the experimental tanks was avoided by placing gel ice packs in the tanks, if necessary; the water temperature was maintained between 17.2 and 20.9 °C, which was within the diurnal fluctuation range of the Agi-gawa River during the study period.

At the beginning of each trial, a randomly selected *Glossosoma* larva was placed at the start line (Fig. 1). The trial was finished when the larva reached either goal line, and then we counted the larva as the individual, which selected between the two types of experimental waters, which flowed through the attained goal line (Fig. 1). If the larva did not reach either goal line within 5 min., we recorded the case as the failed trial. However, only five individuals did not reach either goal line within 5 min., and some of them might have been dead. The failed trials were discarded. Most of individuals reached either goal line in 1–2 min. We randomly performed six experimental combinations with three types of experimental water (control, particulate, and dissolved microalgal cues) and left/right water tanks. An experimental set, which consisted of eight individual larval replicates (*n* = 8), was repeated five times with exchange of the individuals; in all, 240 trials were conducted with 240 individuals.

This study was performed in strict accordance with the recommendations of the Guidelines for the Proper Conduct of Animal Experiments by the Science Council of Japan (Jan 2006). According to the guidelines, a special permission was not required for conducting experiments on invertebrates. All efforts were made to minimize suffering.

### Statistical analysis

Three types of combinations (i.e., control vs. dissolved microalgal cue, control vs. particulate microalgal cue, and dissolved vs. particulate microalgal cue) were used to test the selections by individual larvae of either experimental water by using generalized linear mixing models (GLMMs). The treatment was dealt with as categorical data, and the Poisson distribution was used for the error distribution of GLMMs. The trial replicates were used as the random factors of GLMMs.

The proportion of numbers of *Glossosoma* larvae among the treatments was tested using Newcombe’s test of equal proportions to test the null hypothesis that the proportions in several groups are the same. For considering multiple comparisons for Type I error, the significances of Newcombe’s test among the treatments were fixed using Bonferroni criteria. The difference in periphyton cell abundance of the experimental water was tested using one-way analysis of variance (ANOVA), and post-hoc multiple comparisons were performed using Tukey’s test. For all statistical analyses, *α* = 0.05 was used as the significance criterion. All statistical analyses were performed using R version 2.15.3 (R Development Core Team, 2013).

## Results

Chl *a* concentrations were significantly different among the control, dissolved, and particulate microalgal cue waters (one-way ANOVA, *F* = 20.75, *p* < 0.001). Microalgal abundance was not significantly different between the control and dissolved cue waters (0.8 ± 1.5 and 5.0 ± 2.5 mg Chl *a* m^−3^, respectively; Tukey’s test, *p* > 0.05), and was significantly higher for the water with particulate cues (21.4 ± 8.8 mg Chl *a* m^−3^, *p* < 0.05) than of those of the remaining two treatments. As expected, the water treatment with particulates had the highest Chl *a* concentration.

**Figure 2:**
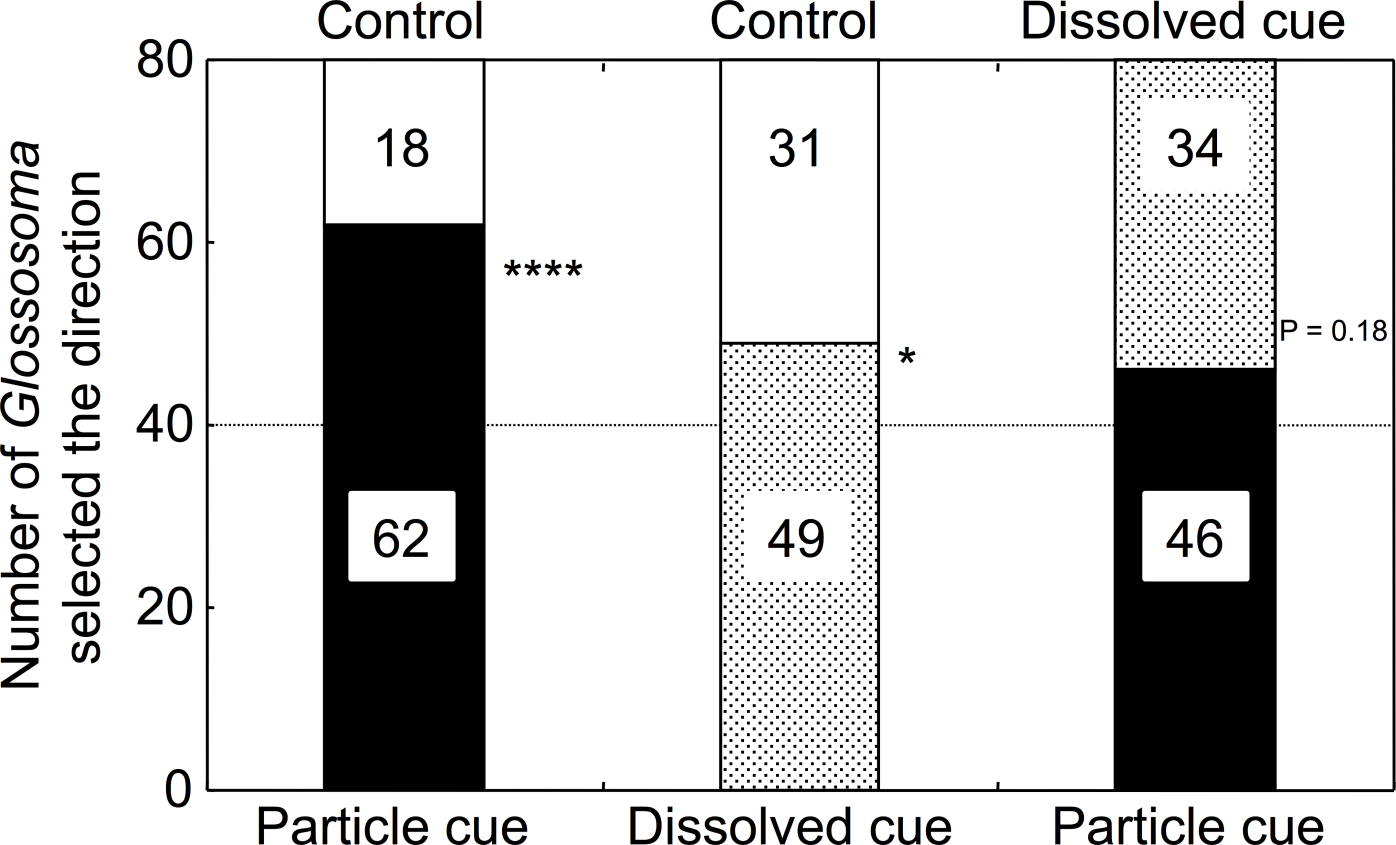
The results for selectivity of the two types of experimental water by the test larvae (*n* = 80 for each combination). ^∗∗∗∗^, ^∗^, and NS show *p* < 0.0001, <0.05, and >0.05 by GLMMs, respectively. All proportions were significantly different among the treatments (Newcombe’s test with Bonferroni criteria, *p* < 0.05).

Significantly higher numbers of *Glossosoma* larvae selected particulate microalgal cue water over control river water (GLMM, *p* < 0.0001; Fig. 2, Appendix S1). In addition, the numbers of larvae that selected dissolved cue water were significantly higher than those that selected control water (*p* = 0.046). However, there was no significant difference in the numbers of larvae that selected either particulate or dissolved cue water (*p* = 0.18). The proportions of *Glossosoma* larvae were significantly different among the treatments (Newcombe’s test of equal proportions with Bonferroni criteria, *p* < 0.05), indicating that the preference for water with particulate cues over control was significantly higher than that for water with dissolved cues.

## Discussion

We found that *Glossosoma* larvae preferred both particulate and dissolved cues (i.e., particulate and chemical cues and chemical cues only, respectively) over the control water. As we hypothesized, both cues were important to determine the foraging direction of the grazer, *Glossosoma* larvae. Preference for particulate cues over the control was significantly higher than that for dissolved cues over the control, indicating that *Glossosoma* larvae responded well when both particulate (i.e., detecting drifting microalgae) and chemical cues were present. *Glossosoma* larvae could probably detect materials drifting from upstream by using their eyes and/or tactile organs. In addition, the present study revealed that the larvae could determine the crawling direction for a suitable food source by using microalgal cues. Accurately determining the crawling direction would be favored by natural selection for increasing feeding both chemical and particulate and facilitating growth (Lamberti & Resh, 1983; Feminella & Resh, 1990; Hart & Robinson, 1990).

The following could be the merits of detecting both chemical and particulate cues from microalgae: (1) Periphyton, especially some diatoms, are known to drift from periphyton mats (Bothwell et al., 1989; Stevenson & Peterson, 1991), and periphytic cells are removed and then suspended by current turbulence in streams and (2) Chemical cues might be transmitted, not only from upstream, but also from other directions. Therefore, grazers would benefit if they can detect particulate cues as well to adjust their search direction, which was initially determined using chemical cues. In streams, the grazers may not be able to detect the chemical and/or particulate cues from upstream, although there are some merits of being able to so. Therefore, even if the habitats with suitable resources are downstream, the grazers cannot detect the cues from these habitats. However, our previous studies (Katano, Doi & Oishi, 2009; Doi, Katano & Kikuchi, 2006) showed that *Glossosoma* grazers could change their direction of movement and habitat use in line with the amount of upstream cues. Thus, cues in stream ecosystems are important to determine *Glossosoma* habitats, even though they cannot detect those downstream.

In this study, we conducted a simple experiment to evaluate the effects of chemical and particulate cues on *Glossosoma* grazers. Although this was an initial step toward understanding the cue communications between producers and grazers, there were a few limitations to this study. First, we did not evaluate the differences in the concentrations of chemical cues for determining the crawling direction. Katano, Doi & Oishi (2009) suggested that an increase in algal cues induces responses in grazer behavior. Thus, increasing microalgal (chemical and particulate) cues should influence crawling direction; for instance, cues arising from smaller algal biomass might not direct grazers toward suitable food patches. Second, in this study, we assumed that grazers would detect both chemical and particulate cues from the experimental water containing dissolved and particulate cues. However, the compositions of microalgal cues (i.e., microalgal cells, materials, species, and dissolved cues) in both the experimental water treatments were not known. Further study is needed to determine the cue materials or algal species that are important for determining the behavior of grazers.

In this study, we found that the grazers could detect and respond to both chemical and particulate cues from microalgae. However, previous studies have shown that prey changed their activity to avoid predation by detecting only the chemical cues from the predators (Kuhara, Nakano & Miyasaka, 2001; Miyasaka et al., 2003; Ferland-Raymond et al., 2010). Both sensitivities for chemical and visual cues would be caused by the difference of their lethality. For example, prey need to rapidly and precisely detect predator chemical cues to effectively avoid the lethal effects of predators (Turner & Montgomery, 2003; Takahara et al., 2012). On the other hand, macroinvertebrate grazers (i.e., predators of periphyton) would not be at a risk of starving to death if they could take turns to reach a patch with abundant periphyton (i.e., prey of grazers). Thus, grazers could efficiently select the direction of crawling toward a suitable periphyton patch by using both chemical and particulate cues. Since microalgae such as diatoms usually drift in natural streams (Bothwell et al., 1989) like particulate microalgal cues, the cues from dissolved matter and particulate matter would provide the grazers with an opportunity to effectively search for food patches.

*Glossosoma* grazers are the primary consumers of periphyton (primary producer) and serve as prey to higher-trophic-level consumers (predators such as fish and/or plecopteran genera). In response to predator cues (i.e., top-down cues), grazers change their movements, drift behavior, and diel periodicity of activity (Kohler & McPeek, 1989; Kuhara, Nakano & Miyasaka, 2001; Miyasaka et al., 2003).

The present study revealed that grazers could also change their behavior based on microalgal cues (i.e., bottom-up cues). Therefore, grazers change their behaviors to maintain a balance between the top-down and bottom-up cues, thereby maximizing fitness by avoiding predation and more efficiently obtaining food resources. Further studies should address the differences in consumer responses between predator and prey cues. This will provide a better understanding of how consumer distribution is regulated by cues from both predators and food sources.

## Supplemental Information

10.7717/peerj.503/supp-1 Appendix S1 Supporting dataset The raw data of the all experiments for combining 3 treatments.
